# Enhancing Patient Outcome Prediction Through Deep Learning With Sequential Diagnosis Codes From Structured Electronic Health Record Data: Systematic Review

**DOI:** 10.2196/57358

**Published:** 2025-03-18

**Authors:** Tuankasfee Hama, Mohanad M Alsaleh, Freya Allery, Jung Won Choi, Christopher Tomlinson, Honghan Wu, Alvina Lai, Nikolas Pontikos, Johan H Thygesen

**Affiliations:** 1 Institute of Health Informatics University College London London United Kingdom; 2 Department of Health Informatics, College of Applied Medical Sciences Qassim University Buraydah Saudi Arabia; 3 UCL Institute of Ophthalmology University College London London United Kingdom

**Keywords:** deep learning, electronic health records, EHR, diagnosis codes, prediction, patient outcomes, systematic review

## Abstract

**Background:**

The use of structured electronic health records in health care systems has grown rapidly. These systems collect huge amounts of patient information, including diagnosis codes representing temporal medical history. Sequential diagnostic information has proven valuable for predicting patient outcomes. However, the extent to which these types of data have been incorporated into deep learning (DL) models has not been examined.

**Objective:**

This systematic review aims to describe the use of sequential diagnostic data in DL models, specifically to understand how these data are integrated, whether sample size improves performance, and whether the identified models are generalizable.

**Methods:**

Relevant studies published up to May 15, 2023, were identified using 4 databases: PubMed, Embase, IEEE Xplore, and Web of Science. We included all studies using DL algorithms trained on sequential diagnosis codes to predict patient outcomes. We excluded review articles and non–peer-reviewed papers. We evaluated the following aspects in the included papers: DL techniques, characteristics of the dataset, prediction tasks, performance evaluation, generalizability, and explainability. We also assessed the risk of bias and applicability of the studies using the Prediction Model Study Risk of Bias Assessment Tool (PROBAST). We used the PRISMA (Preferred Reporting Items for Systematic Reviews and Meta-Analyses) checklist to report our findings.

**Results:**

Of the 740 identified papers, 84 (11.4%) met the eligibility criteria. Publications in this area increased yearly. Recurrent neural networks (and their derivatives; 47/84, 56%) and transformers (22/84, 26%) were the most commonly used architectures in DL-based models. Most studies (45/84, 54%) presented their input features as sequences of visit embeddings. Medications (38/84, 45%) were the most common additional feature. Of the 128 predictive outcome tasks, the most frequent was next-visit diagnosis (n=30, 23%), followed by heart failure (n=18, 14%) and mortality (n=17, 13%). Only 7 (8%) of the 84 studies evaluated their models in terms of generalizability. A positive correlation was observed between training sample size and model performance (area under the receiver operating characteristic curve; *P*=.02). However, 59 (70%) of the 84 studies had a high risk of bias.

**Conclusions:**

The application of DL for advanced modeling of sequential medical codes has demonstrated remarkable promise in predicting patient outcomes. The main limitation of this study was the heterogeneity of methods and outcomes. However, our analysis found that using multiple types of features, integrating time intervals, and including larger sample sizes were generally related to an improved predictive performance. This review also highlights that very few studies (7/84, 8%) reported on challenges related to generalizability and less than half (38/84, 45%) of the studies reported on challenges related to explainability. Addressing these shortcomings will be instrumental in unlocking the full potential of DL for enhancing health care outcomes and patient care.

**Trial Registration:**

PROSPERO CRD42018112161; https://tinyurl.com/yc6h9rwu

## Introduction

### Background

In recent decades, there has been a rapid growth in the use of electronic health records (EHRs) in health care systems, making them an important tool for health care workers and allowing for secondary use for research purposes. Structured EHRs contain temporal records of patient visits, incorporating various clinical data such as diagnosis codes, procedures, and laboratory test results, all of which may help researchers in predicting patient outcomes. Patient timelines can be organized based on diagnosis codes and their corresponding visit times, allowing deep learning (DL) algorithms to model and understand disease progression, with the time between visits representing the speed of disease progression. Using sequential diagnostic data from EHRs in this manner is a promising avenue for DL-based studies, but the degree to which this information has been used in published studies and its benefits has not yet been explored in the context of a systematic review, which is what this study sets out to do.

Classical machine learning (ML) techniques that require feature selection can be applied to include diagnosis codes as a binary feature for outcome prediction; for example, a recent study applied ML-based algorithms (logistic regression, extreme gradient boosting, and random forest) to identify cardiomyopathy [1]. However, traditional ML approaches cannot take full advantage of structured EHR data due to four key challenges:

1. Feature selection—manual feature selection, which requires medical knowledge from professional health care workers, is a time-consuming task and an expensive process.
2. High dimensionality—models suffer from a high-dimensional input representation due to the vast number of medical codes available (eg, Medical Information Mart for Intensive Care [MIMIC]-IV includes >15,000 unique *International Classification of Diseases, Tenth Revision* [ICD-10], codes that appear in the patient records) [2].
3. Hierarchy—the hierarchical structure of diagnosis codes may represent relationships between similar disease categories, but this information is ignored by traditional ML-based approaches.
4. Temporality—the majority of traditional ML techniques struggle to effectively capture information contained in the temporal chronological sequence of patients’ medical history, where the time between consecutive visits may vary in length from a few days to numerous months. Significant predictive insights may be hidden in the temporal intervals and sequence of diagnosis codes in a patient’s evolving medical history because deterioration or improvement in outcomes may follow specific patterns and frequencies of interactions with the health care system [3-5].

To comprehensively uncover and understand the impact of these intricate temporal and sequential relationships within the data, advanced DL methods are essential.

DL approaches have been applied previously in the health care domain. Systematic reviews show a good progression in DL-based algorithms for various medical data types, such as clinical notes [6], medical images [7], and physiological signals [8]. DL emerges as a solution to overcome the aforementioned limitations of traditional ML for the following reasons: (1) DL functions as an end-to-end system that can automatically uncover an association between input and output with minimal need for feature engineering or domain expertise; (2) DL models can generate an effective embedding space to cope with the high-dimensional problem (eg, a study demonstrated the effectiveness of an autoencoder in transforming RNA sequence data with approximately 20,000 features into a low-dimensional representation with approximately 1000 features, achieving high classification performance [9]); (3) some DL techniques, such as graph neural networks (GNNs), have been shown to give a good representation of hierarchical data [10]; and (4) to deal with temporal information, long short-term memory (LSTM) and temporal convolutional neural network (CNN) models have been adapted widely for complex sequential information in health care (eg, an LSTM model has been shown to be able to achieve a good performance in analyzing information from high-volume regular sequences such as intensive care unit [ICU] monitoring data [11]).

Although many DL techniques are well suited for hierarchical and time-series data, they face challenges in handling sequential diagnosis codes. In EHRs, diagnosis codes occur at irregular time intervals, reflecting the varying times between medical events, that is, some patients may have multiple visits within the same week, while for others, there may be months or years between visits. This irregularity complicates analysis but is also a source of information because it may capture the rapid or slow progression of conditions. Moreover, diagnosis codes require an embedding layer before being processed by a DL model, unlike continuous values from ICU monitors, which can be directly processed. Other challenges with DL are the need for extensive datasets and concerns about explainability [12]. The performance of existing DL techniques depends on the volume and quality of the training dataset, which, in the field of health data science, may be problematic because large-scale datasets may not be available due to privacy concerns. This can also contribute to the generalizability issue because models may perform well on internal training and test datasets but perform poorly on independent external data sources. Moreover, DL model predictions can sometimes be unclear to clinicians, and there is a need to explain the main factors contributing to the model’s output. Therefore, it becomes a significant challenge for researchers to use special DL techniques for outcome prediction by using sequential diagnosis codes. This systematic review will comprehensively explore these challenges and the approaches used to deal with them in the published literature.

To date, several reviews have analyzed DL methods trained on EHR data [13-16]. Various kinds of EHR data for DL-based algorithms have been surveyed: (1) structured data (diagnosis codes, medication codes, procedure codes, laboratory test results, and vital signs) and (2) unstructured data (clinical notes, medical images, and physiological signals). However, none of the reviews primarily focused on the use of sequential diagnostic data in DL for outcome prediction. Moreover, none of them reported on the inclusion of external validation, which can be problematic because models are applied to different data distributions. Many questions remain unanswered, such as common DL techniques, types of diagnosis codes, additional features (eg, time between visits, demographic data, and medications), dataset characteristics, prediction tasks, generalizability, and explainability.

### Objectives

We conducted a systematic review to answer these questions and investigate the current state of DL in the context of outcome prediction using sequential diagnostic information. By summarizing the research in this area, our review can help guide future DL-based prediction studies by identifying current research gaps and challenges. The main objective of this systematic review was to identify and summarize existing DL studies that use sequential diagnosis codes as key predictors of patient outcomes. In addition, this study investigates the challenges of generalizability and explainability in these predictive models.

## Methods

### Definition

In this systematic review, we defined sequential diagnosis codes as medical codes (eg, Systemized Nomenclature of Medicine–Clinical Terms; *International Classification of Diseases, Ninth Revision*; and *ICD-10* codes) assigned to patients to represent their visits within the health care system. This review examined various categories of DL algorithms, including recurrent neural networks (RNNs), LSTM models, CNNs, transformer-based models, and GNNs, in addition to some techniques such as time-awareness and attention mechanisms. No restrictions were placed on study outcomes, which included mortality, hospitalization status, and onset of disease (eg, hypertension, diabetes, heart attack, stroke, and cancer).

### Search Strategy

As our review combines knowledge from both health care and engineering, we sought to identify all relevant studies in both domains using 4 databases: PubMed, Embase, IEEE Xplore, and Web of Science. In addition, we conducted a manual search of the reference lists of the included studies to identify additional relevant articles. We searched the databases up to May 15, 2023. To promote transparency and prevent duplication, the study protocol was registered in PROSPERO (CRD42023434032).

We used 4 main groups of keywords centered around DL techniques, EHRs, sequence, and prediction. The literature search included the following search terms: (“deep learning” OR “RNN” OR “LSTM” OR “CNN” OR “transformer” OR “BERT” OR “time attention” OR “attention based” OR “graph neural network”) AND (“electronic health records” OR “EHRs” OR “electronic health record” OR “EHR” OR “electronic medical record” OR “EMR” OR “electronic medical records” OR “EMRs”) AND (“longitudinal” OR “visit” OR “sequential” OR “sequence” OR “temporal”) AND (“risk” OR “predictions” OR “prediction” OR “patient outcome” OR “prognosis”). The search query returned the same set of results as those obtained using the built-in search functionality of the literature databases.

### Inclusion and Exclusion Criteria

This systematic review followed the Population, Intervention, Comparison, and Outcomes framework to identify and select articles in the databases [17]. The population included patients of all ages in EHR databases, the intervention involved DL-based methods for sequential diagnosis codes, the comparison was between different algorithms, and the outcome was model performance.

We included all studies using DL algorithms to predict patient outcomes by training the models on sequential or longitudinal diagnosis codes, as defined in the aforementioned search terms. We excluded review articles and non–peer-reviewed papers. In addition, we excluded papers that primarily dealt with other nondiagnostic EHR data types, including physiological signals, clinical notes, and medical images. To reduce bias, 2 reviewers independently screened all studies. Any discrepancies between the reviewers were resolved through discussion to reach a consensus. The level of agreement between the reviewers was assessed using the Cohen κ coefficient.

### Extraction and Analysis

We used the PRISMA (Preferred Reporting Items for Systematic Reviews and Meta-Analyses) checklist [18] (Multimedia Appendix 1) to report our findings. We evaluated the following aspects in the included papers: DL techniques, characteristics of the datasets, prediction tasks, and performance evaluation. For each study, we selected either the novel, proposed technique or the best-performing technique as the main DL model architecture. The findings are presented using plots, figures, and tables.

This review followed the suggestions of a previous study [19] to assess the generalizability of the applied models. Generalizability was evaluated based on the potential applicability of outcome predictive models beyond their original development context, focusing on 3 key aspects:

1. Demographic validation, which investigates the model’s adaptability to distinct clinical contexts, including disparities related to sex or ethnicity and variations in age groups
2. Temporal validation, which focuses on assessing the model’s performance over time within its original development environment
3. Geographic validation, which explores the model’s capacity to extend its utility beyond its original development setting to different locations, institutions, or geographic contexts

In addition, we evaluated the explainability of the model in each included study.

### Risk-of-Bias and Quality Assessment

The Prediction Model Study Risk of Bias Assessment Tool (PROBAST) [20] was used to evaluate both the risk of bias (ROB) and the applicability of the best-performing DL models in the included studies. ROB was evaluated based on a set of 20 questions categorized into 4 domains: participants, predictors, outcome, and analysis. Applicability was evaluated via a main question for each of the following 3 domains: participants, predictors, and outcomes. Each domain was rated as having low, unclear, or high ROB. If multiple models were reported in a study, only the model with the highest area under the receiver operating characteristic curve (AUROC) and *F*_1_-score was evaluated. One reviewer conducted the PROBAST assessment for all included studies.

## Results

### Study Selection

**Figure 1:**
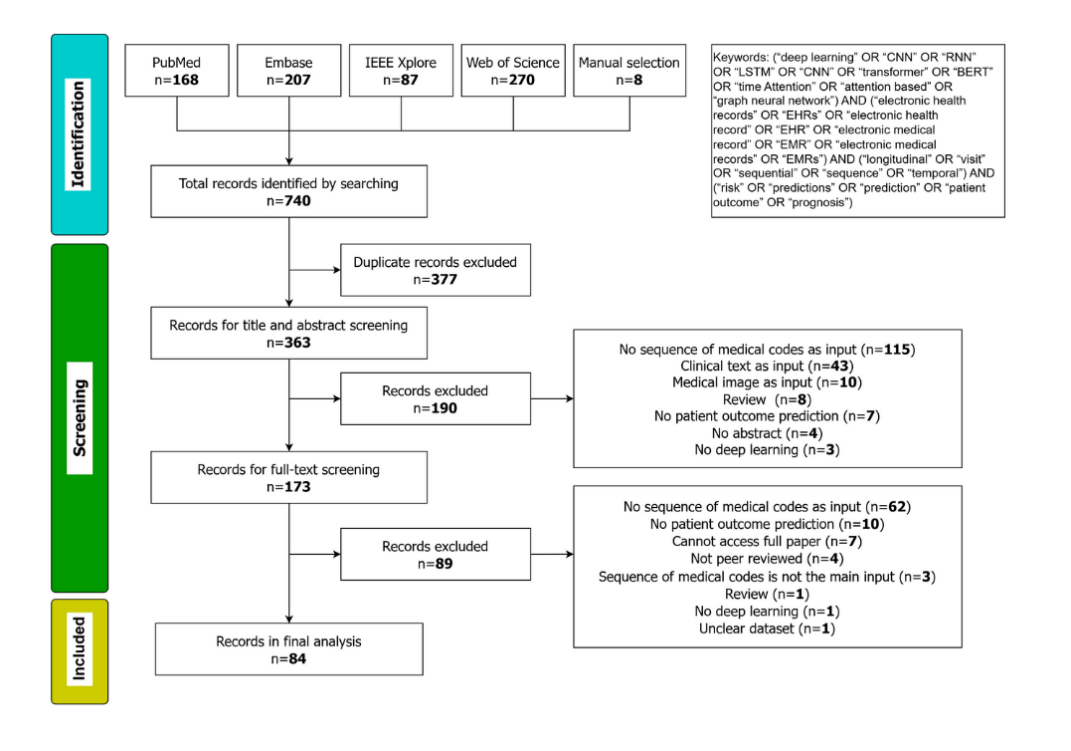
PRISMA (Preferred Reporting Items for Systematic Reviews and Meta-Analyses) flow diagram of the search and screening results.

Figure 1 presents the PRISMA diagram of the search and screening results. Initially, our search identified 740 records, of which 377 (50.9%) duplicates were removed. The screening process consisted of 2 stages: title and abstract screening, followed by full-text screening. During the title and abstract screening, we assessed the study aim, objectives, and methods to determine whether each paper fell within the scope of our review. Ultimately, 84 (11.4%) of the initially identified 740 articles were included in the final analysis. The agreement between reviewers had a Cohen κ coefficient of 0.65, indicative of a moderate agreement [21]. All included studies are listed in Table 1 and Multimedia Appendix 2 [22-105].

**Table 1:** Summary list of included studies, highlighting deep learning (DL) approaches, prediction tasks, additional features, included time, performance evaluation, risk of bias (ROB), and concern of applicability (COA).

Study; year	DL approach	Prediction task	Additional features	Included time	Performance evaluation	ROB	COA
Miotto et al [22]; 2016	Autoencoders	New onset of disease	Demographic data, medications, procedures, laboratory test results, and clinical text	No	Accuracy, AUROC^a^, and *F*_1_-score	Unclear	Low
Nguyen et al [23]; 2016	CNN^b^	Readmission	No	Yes (as month)	Accuracy	High	Unclear
Choi et al [24]; 2016	GRU^c^	Next-visit diagnosis	Medications and procedures	Yes	Recall	High	Unclear
Pham et al [25]; 2016	LSTM^d^	Readmission	Procedures, medications, and admission type	Yes (as day)	*F*_1_-score	High	Unclear
Choi et al [26]; 2016	RNN^e^	Heart failure	Medications and procedures	Yes	AUROC	High	Low
Ma et al [27]; 2017	GRU	Next-visit diagnosis	Procedures	No	Accuracy	High	Unclear
Choi et al [28]; 2017	RNN	Next-visit diagnosis and heart failure	No	No	Accuracy and AUROC	High	Low
Sha and Wang [29]; 2017	GRU	Mortality	No	No	AUROC, *F*_1_-score, and MCC^f^	High	Unclear
Choi et al [30]; 2017	GRU	Heart failure	Medications and procedures	Yes	AUROC	High	Low
Suo et al [31]; 2017	CNN	Diabetes, obesity, and COPD^g^	No	No	Accuracy	High	Unclear
Amirkhan et al [32]; 2017	LSTM	Colorectal cancer	Medications and laboratory test results	Yes (as day)	AUROC	Low	Low
Lei et al [33]; 2018	RNN	Mortality and comorbidity	Demographic data, laboratory test results, medications, and procedures	No	Accuracy, AUC^h^, and *F*_1_-score	High	Low
Park et al [34]; 2018	RNN	CVDs^i^	Medications	Yes	Sensitivity, specificity, positive predictive value, *F*_1_-score, and AUROC	High	Unclear
Bai et al [35]; 2018	RNN	Next-visit diagnosis	Procedures	Yes	Accuracy and *F*_1_-score	High	High
Choi et al [36]; 2018	GRU and CNN	Heart failure and next-visit diagnosis	Medications and procedures	No	AUROC and AUPRC^j^	High	Low
Qiao et al [37]; 2018	RNN	Next-visit diagnosis	Procedures	Yes	Recall and AUROC	High	Unclear
Wang et al [38]; 2018	GRU	Next-visit diagnosis	Medications and demographic data	No	Precision and *F*_1_-score	High	Unclear
Ma et al [39]; 2018	LSTM	Next-visit diagnosis	No	Yes (as week)	Accuracy and precision	High	Unclear
Zhang et al [40]; 2018	GRU	Hospitalization	Demographic data	No	AUROC, sensitivity, specificity, and *F*_2_-score	Unclear	Low
Jin et al [41]; 2018	LSTM	Heart failure	No	No	AUROC, AUPRC, and *F*_1_-score	High	Unclear
Guo et al [42]; 2019	LSTM	Next-visit diagnosis	Medications	No	Accuracy, recall, precision, and *F*_1_-score	High	Unclear
Lin et al [43]; 2019	LSTM	Readmission	Vital signs and demographic data	Yes (as hour)	AUC and recall	Unclear	Unclear
Wang et al [44]; 2019	RNN	Next-visit diagnosis	Physical symptoms and medications	No	Precision	High	Unclear
Gao et al [45]; 2019	GRU	Next-visit diagnosis	Demographic data	No	Recall and precision	High	Unclear
Ma et al [46]; 2019	GNN^k^	Next-visit diagnosis	No	No	Precision and accuracy	High	Unclear
AlSaad et al [47]; 2019	LSTM	Asthma	No	No	AUROC	High	Low
Zhang et al [48]; 2019	LSTM and CNN	MCI^l^, Alzheimer disease, and Parkinson disease	No	No	AUROC and *F*_1_-score	High	Unclear
Ashfaq et al [49]; 2019	LSTM	Readmission	Human-derived features, procedures, medications, and laboratory test results	No	AUROC and *F*_1_-score	High	Unclear
Ruan et al [50]; 2019	RNN-DAE^m^	Mortality	Medications, laboratory test results, and demographic data	No	AUROC	High	Low
Huang et al [51]; 2019	LSTM	Mortality	Laboratory test results	No	Accuracy, AUROC, and AUPRC	High	Low
Xiang et al [52]; 2019	LSTM	Heart failure	Medications and procedures	Yes	AUROC	High	Unclear
Gupta et al [53]; 2019	LSTM	Obesity	Demographic data, conditions, procedures, medications, and measurement	Yes (as month)	AUROC	High	Low
Shi et al [54]; 2020	LSTM	Mortality	Demographic data	No	Accuracy, recall, and *F*_1_-score	High	Low
Li et al [55]; 2020	Transformers	Next-visit diagnosis and new onset of disease	Age	No	AUROC and precision	Low	Low
Peng et al [56]; 2020	Transformers	Readmission and next-visit diagnosis	Procedures	Yes (as day)	AUPRC and precision	Unclear	Low
Almog et al [57]; 2020	LSTM	Fracture	Demographic data	No	AUROC, recall, specificity, precision, and AUPRC	High	Unclear
Luo et al [58]; 2020	Transformers	COPD, heart failure, and kidney disease	No	Yes	Accuracy, precision, recall, *F*_1_-score, and AUROC	High	Unclear
Zhang et al [59]; 2020	Transformers	Next-visit diagnosis, heart failure, diabetes, and chronic kidney disease	No	No	Precision, accuracy, and AUROC	High	Unclear
Rongali et al [60]; 2020	LSTM	Mortality	Procedures, laboratory test results, medications, clinical events, and demographic data	No	AUROC	Unclear	Unclear
Ye et al [61]; 2020	Transformers and CNN	Heart failure, kidney disease, and dementia	No	No	AUROC, precision, recall, and *F*_1_-score	Unclear	Unclear
Zeng et al [62]; 2020	Transformers	Next-visit diagnosis	Procedures and medications	Yes	Recall	High	Low
An et al [63]; 2020	LSTM	Next-visit diagnosis	Medications and procedures	No	Jaccard similarity score, AUPRC, recall, and *F*_1_-score	High	Low
Kabeshova et al [64]; 2020	LSTM and GRU	Relapse of urinary problems	Medications, procedures, and length of stay	Yes (as day)	Precision, AUROC, and AUPRC	Low	Low
Darabi et al [65]; 2020	Transformers	Readmission, mortality, length of stay, and next-visit diagnosis	Demographic data and clinical text	No	AUROC and AUPRC	Unclear	Unclear
An et al [66]; 2021	LSTM	Risk of CVDs	Demographic data, patient type, hospital visit times, and surgery history	No	Recall, precision, *F*_1_-score, and AUROC	Low	Low
Meng et al [67]; 2021	Transformers	Depression	Demographic data and visit	No	AUROC and AUPRC	High	Low
Rasmy et al [68]; 2021	Transformers	Heart failure and cancers	No	No	AUROC	Low	Low
Ju et al [69]; 2021	CNN	Diabetes and heart failure	Vital signs, demographic data, medications, allergies, and smoking status	Yes (as day)	Accuracy, and AUROC	Unclear	Unclear
Harerimana et al [70]; 2021	GRU	Length of stay and mortality	Demographic data, free-text diagnosis, procedures	No	Accuracy, AUROC, AUPRC, *F*_1_-score, and linear weighted κ	High	Unclear
Ningrum et al [71]; 2021	CNN	Risk of OA^n^ knee	Demographic data and medications	Yes (as week)	AUROC, sensitivity, specificity, and precision	High	Low
Florez et al [72]; 2021	Transformers	Next-visit diagnosis	No	No	Recall, precision, and AUC	High	Unclear
Pham et al [73]; 2021	Transformers	Cardiac complication risk	Demographic data	Yes (as day)	AUROC	High	Low
Boursalie et al [74]; 2021	Transformers	Next-visit diagnosis	Demographic data, medicine, and treatment	Yes	Precision and recall	High	Unclear
Dong et al [75]; 2021	LSTM	Opioid use disorder	Procedures, laboratory test results, medications, clinical events, and demographic data	No	AUROC, precision, recall, and *F*_1_-score	Low	Low
Kwak et al [76]; 2021	GRU	CVDs	Medication and demographic data	No	AUROC and AUPRC	Low	Low
Sun et al [77]; 2021	GRU	Mortality, readmission, sepsis, and heart failure	No	Yes	AUROC, AUPRC, and accuracy	High	Low
Men et al [78]; 2021	LSTM	Next-visit diagnosis	Disease types and demographic data	Yes	AUROC, precision, recall, and *F*_1_-score	High	Low
Shi et al [79]; 2021	CNN	Next-visit diagnosis	Medications	No	Accuracy	High	Unclear
Lu et al [80]; 2021	Multilayer perceptron	Next-visit diagnosis and heart failure	No	No	AUROC and *F*_1_-score	High	Low
Peng et al [81]; 2021	Transformers	Next-visit diagnosis	No	Yes	Accuracy	High	Unclear
An et al [82]; 2021	Bi-LSTM-CNN^o^	CVDs	Medications, laboratory test results, and examination	Yes	Recall, precision, *F*_1_-score, and AUROC	High	Unclear
Poulain et al [83]; 2021	Transformers	Risk of CVDs	Demographic data	Yes (as age)	MSE^p^	High	Unclear
Pang et al [84]; 2021	Transformers	Heart failure, mortality, diabetes, and hospitalization	Medications, procedures, and age	Yes (as month)	AUROC and AUPRC	Unclear	Low
Rao et al [85]; 2022	Transformers	Heart failure	Medications, age, and calendar year	Yes (as year)	AUROC and AUPRC	Low	Low
Du et al [86]; 2022	LSTM	Mortality	No	No	Accuracy, AUROC, and *F*_1_-score	High	Low
De Barros and Rodrigues [87]; 2022	LSTM	Next-visit diagnosis	No	No	Recall, precision, AUROC, and *F*_1_-score	High	Unclear
Liu et al [88]; 2022	Transformers	Next-visit diagnosis and mortality	Medications, laboratory test results, clinical events, and demographic data	Yes	AUROC, AUPRC, and recall	High	Low
Yang et al [89]; 2022	LSTM	Mortality	Admission type	Yes (as day)	AUC, precision, recall, and *F*_1_-score	High	Low
Chen et al [90]; 2022	Transformers	Next-visit diagnosis and new onset of disease	Procedures	Yes	Precision and recall	Low	Low
Sun et al [91]; 2022	GRU	Next-visit diagnosis	No	No	*F*_1_-score and recall	High	Unclear
Yu et al [92]; 2022	Logical perception	Next-visit diagnosis and mortality	Medications and procedures	No	Accuracy, precision, recall, and *F*_1_-score	High	Low
Niu et al [93]; 2022	GRU	Mortality	Demographic data, procedures, medications, and vital signs	No	AUROC, *F*_1_-score, precision, sensitivity, and specificity	Unclear	Unclear
AlSaad et al [94]; 2022	RNN	Emergency visit	No	No	AUROC, AUPRC, and *F*_1_-score	Low	Low
Gerrard et al [95]; 2022	Transformers	Next-visit diagnosis and readmission	No	No	AUROC and *F*_1_-score	High	Low
AlSaad et al [96]; 2022	RNN	Preterm birth	Procedures, medications, and laboratory test results	No	AUROC, AUPRC, sensitivity, and specificity	Unclear	Low
Ramchand et al [97]; 2022	RNN	Hospitalization for COVID-19 infection	Demographic data	Yes	AUROC, *F*_1_-score, sensitivity, and specificity	High	Low
Andjelkovic et al [98]; 2022 [98]	LSTM and GRU	Lung cancer, breast cancer, cervix uteri cancer, and liver cancer	No	No	Accuracy, AUROC, recall, specificity, precision, and *F*_1_-score	High	Low
Yu et al [99]; 2022	LSTM	Mortality	Medications and procedures	No	Precision, recall, AUROC, accuracy, and *F*_1_-score	High	Unclear
Li et al [100]; 2022	Transformers	Heart failure, stroke, and coronary heart disease	Age	Yes (as age)	AUROC and precision	Low	Low
Li et al [101]; 2023	Transformers	Heart failure, diabetes, chronic kidney disease, and stroke	Medications, procedures, laboratory test results, blood pressure, drinking status, smoking status, and BMI	No	AUROC and AUPRC	Low	Low
Dong et al [102]; 2023	LSTM	Opioid overdose	Medications, laboratory test results, clinical events, and demographic data	No	precision, recall, *F*_1_-score, and AUROC	Low	Low
Guo et al [103]; 2023	Transformers	Mortality, long length of stay, readmission, and ICU^q^ admission	Demographic data, laboratory test results, procedures, and medications	Yes	AUROC and AUPRC	Unclear	Unclear
Liang and Guo [104]; 2023	Transformers	Heart failure	Demographic data, laboratory test results, procedures, and medications	No	Accuracy, AUROC, and *F*_1_-score	High	Low
Lee et al [105]; 2023	CNN	Psoriatic arthritis	Medications	Yes	AUROC, sensitivity, specificity, PPV^r^, and NPV^s^	High	Low

^a^AUROC: area under the receiver operating characteristic curve.

^b^CNN: convolutional neural network.

^c^GRU: gated recurrent unit.

^d^LSTM: long short-term memory.

^e^RNN: recurrent neural network.

^f^MCC: Matthews correlation coefficient.

^g^COPD: chronic obstructive pulmonary disease.

^h^AUC: area under the curve.

^i^CVD: cardiovascular disease.

^j^AUPRC: area under the precision-recall curve.

^k^GNN: graph neural network.

^l^MCI: mild cognitive impairment.

^m^RNN-DAE: recurrent neural network–based denoising autoencoder.

^n^OA: osteoarthritis.

^o^Bi-LSTM-CNN: bidirectional long short-term memory–convolutional neural network.

^p^MSE: mean squared error.

^q^ICU: intensive care unit.

^r^PPV: positive predictive value.

^s^NPV: negative predictive value.

### DL Techniques

**Figure 2:**
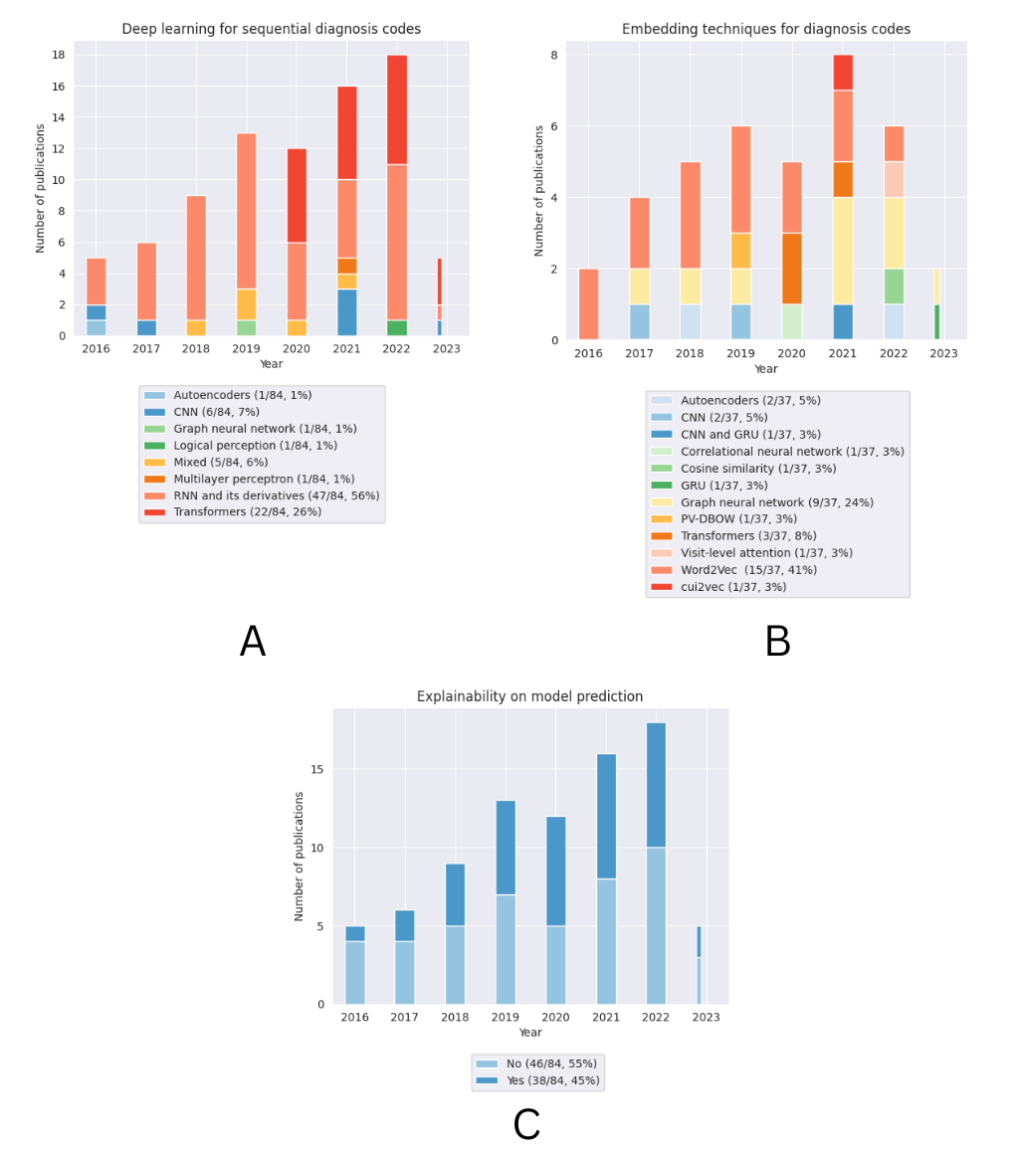
The publication pattern in terms of (A) the number of deep learning models, (B) embedding techniques, and (C) explainability. The thin bar representing 2023 reflects the partial year of data because the search only extended to May 2023. CNN: convolutional neural network; GRU: gated recurrent unit; PV-DBOW: paragraph vector–distributed bag of words; RNN: recurrent neural network.

We analyzed 84 DL models from the included studies. Among these 84 models, the most commonly applied DL technique for learning sequential diagnosis codes was RNNs and their derivatives (n=47, 56%), followed by transformers (n=22, 26%), which have been regularly applied in studies since their introduction in 2017 (Figure 2). Among the 38 studies that used embedding techniques to represent diagnostic data, the most frequently used embedding method was Word2Vec (n=15, 39%), followed by GNNs (n=9, 24%) and transformers (n=3, 8%). More than half of the 84 studies presented their input feature as a sequence of visit embeddings (n=45, 54%), followed by a sequence of diagnosis codes (n=24, 29%), a sparse matrix (n=10, 12%), and a mixed representation (n=5, 6%).

### Dataset Characteristics

Across 125 training datasets, the median sample size was 29,256 (IQR 92,572; range 1095-5,231,614) patients. The most frequently used datasets (82/125, 65.6%) originated from the United States. Moreover, there is an increase in sample size for training models (Figure S1 in Multimedia Appendix 3). The publicly available MIMIC-III dataset (31/125, 24.8%) was the most popular, followed by the Clinical Practice Research Datalink (11/125, 8.8%), Cerner Health Facts (8/125, 6.4%), Sutter Health (5/125, 4%), and MIMIC-IV (3/125, 2.4%; Figure S2 in Multimedia Appendix 3). The most frequently used coding system was *International Classification of Diseases, Ninth Revision* (82/125, 65.6%), followed by *ICD-10* (30/125, 24%). The most frequently incorporated additional feature in the 84 studies was medications (n=38, 45%), followed by demographic data (n=33, 39%), procedures (n=29, 35%), laboratory test results (n=15, 18%), and clinical events (n=4, 5%; Figure S3 in Multimedia Appendix 3). Some studies (35/84, 42%) integrated time information into their DL models for understanding patient prognosis.

### Prediction Tasks

**Figure 3:**
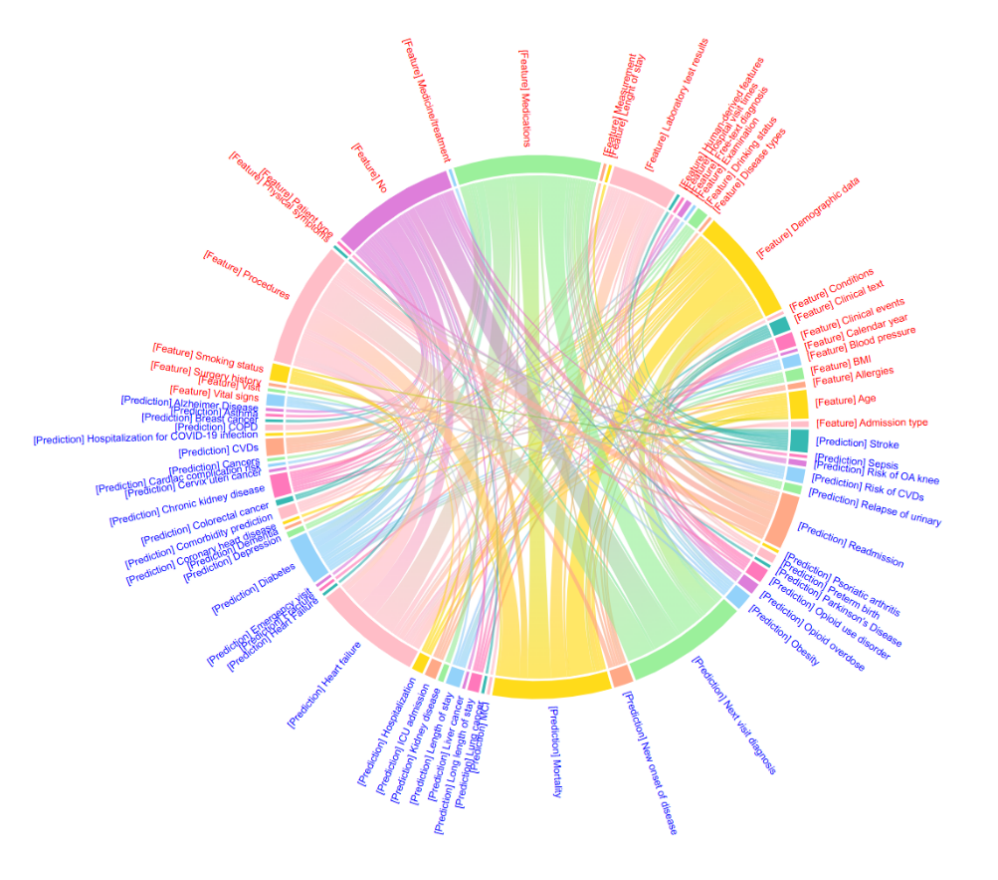
Chord diagram showing the relationship between features (red) and predictions (blue), derived from the “Additional features” and “Prediction task” columns in Table 1, respectively. In the chord diagram, “No” represents the absence of any features; “Visit” refers to the number of times a patient visited a health care provider; “Human-derived features” refers to features extracted from human input, such as manually recorded clinical observations or patient-reported outcomes; “Measurement” represents objective quantifications, such as laboratory test results, vital signs, and other instrument-based evaluations; “Medications” refers to prescribed drugs; “Medicine/treatment” is a broader term that includes medications and surgical procedures; “Calendar year” denotes the year of a patient’s clinic visit; and “Conditions” refers to medical conditions.

The highest frequency of predicted outcome variables were patient trajectory (n=51, 39.8%), cardiovascular disease and risks (n=33, 25.8%), admission (n=15, 11.7%), neurological diseases (n=6, 4.7%), and malignancy (n=6; 4.7%). The main subgroups of prediction tasks were next-visit diagnosis (n=30, 23.4%), heart failure (n=18, 14.1%), and mortality (n=17, 13.3%). Figure 3 presents the chord diagram illustrating the relationships between features and outcome predictions. The widest band is the connection between medications and next-visit diagnosis (10/263, 3.8%).

### Performance Evaluation

A variety of model performance metrics were reported across the 84 included studies. The best DL model performance in each study was reported using AUROC (41/84, 49%), *F*_1_-score (25/84, 30%), area under the precision-recall curve (13/84, 15%), precision (16/84, 19%), and recall (14/84, 17%). The relationship between sample size, the number of features, and AUROC was examined (Figure S4 in Multimedia Appendix 3). A statistically significant relationship was found between sample size and AUROC (*P*=.02), indicating that changes in sample size have a notable impact on AUROC. However, there was no statistically significant relationship between the number of features and AUROC.

### Generalizability and Explainability

An assessment of generalizability with external validation was uncommon among the included studies. Overall, only 7 (8%) of the 84 studies evaluated generalizability across ≥1 of the following categories: demographic validation (n=3, 43%) [39,48,55], temporal validation (n=2, 29%) [100,103], and geographic validation (n=4, 57%) [24,39,68,100]. Regarding explainability, less than half of the studies (38/84, 45%) incorporated a mechanism to interpret their predictions. The publication pattern in terms of explainability is shown in Figure 2.

### ROB and Concern of Applicability

**Figure 4:**
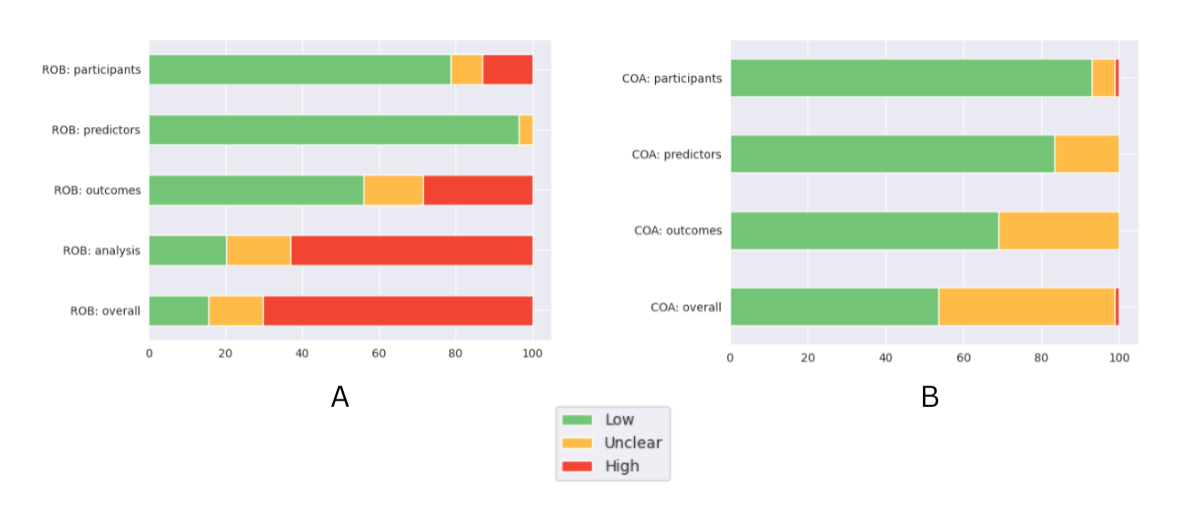
The Prediction Model Study Risk of Bias Assessment Tool (PROBAST) results: (A) risk of bias (ROB) and (B) concern of applicability (COA).

Overall, the included studies had a high ROB (59/84, 70%), which was mainly driven by high ROB in the analysis domain (53/84, 63%). The main reason for high ROB in the analysis domain was the imbalance between the number of patients and their outcomes. Our assessment found low ROB in the participant (66/84, 79%) and predictor (81/84, 96%) domains due to broad inclusion criteria in general and similar predictor definitions across all participants, respectively. Within the applicability assessment, 3 domains were evaluated: participants, predictors, and outcomes. Overall, 45% (38/84) of the included studies showed either unclear or high concern of applicability because the characteristics of most included studies were not clearly mentioned. The full results of the ROB and applicability assessment are shown in Figure 4.

## Discussion

### Model Architecture

This systematic review offers insights into the contemporary DL approaches used to model patients’ diagnostic history to predict outcomes. We explored 84 studies that met our inclusion criteria in this research area. The application of DL for advanced modeling of sequential medical codes is a rapidly growing research area. This is evident from the increasing number of publications found each year in this review. Considering the escalating interest in DL, there have been obvious publication patterns since 2016. After the emergence of bidirectional encoder representations from transformers (BERT) at the end of 2018 [106], transformer models have been increasingly used for sequential diagnosis codes, as reflected in the literature. More recently, there has been a prominent and highly successful showcase of transformers, with OpenAI’s ChatGPT with GPT-3.5 and ChatGPT with GPT-4 [107] serving as prime examples of their capabilities.

Diagnosis code and language share some similar aspects. We can consider diagnosis codes as words and code sequences in a medical history as sentences. Typically, DL models designed for diagnosis codes aim to capture relationships between diagnosis codes within patient visits and across patient visits, which is similar to natural language processing (NLP) approaches that learn connections between words within sentences and across different sentences. Numerous studies in our review applied recent NLP techniques, such as RNNs and transformers, to sequential diagnosis codes. The main difference between diagnosis codes within patient visits and words within sentences is the irregular time interval between medical events. To accommodate this difference, many studies adjust the original DL algorithms to make them suitable for sequential diagnosis codes. Some examples include RETAIN [26], Dipole [27], EHAN [34], Timeline [35], Patient2Vec [40], DeepRisk [108], COAM [42], CLOUT [60], HAN [70], IoHAN [86], AttentionHCare [87], DeepMPM [89], and tBNA-PR [104], all of which use attention mechanisms and show superior performance compared to models without attention mechanisms.

Although RNNs and their derivatives made up the majority of the models (47/84, 56%) used for patient outcome prediction, several studies (15/84, 18%) showed that transformers have superior performance [55,56,59,62,67,72,73,81,83-85,88,90,95,104]. The transformer model consists of a multihead self-attention unit that computes in parallel. A crucial part of this architecture is the positional encoder, which enables transformers to understand the order and adjacency of information, in a similar way to CNNs and RNNs, respectively. Evidence from 2 (2%) of the 84 studies included in this review shows that positional embedding can improve model performance [55,64]. Moreover, models that combine transformers and RNNs have been introduced; for example, the medical BERT model combined with bidirectional gated recurrent unit improved the AUROC of the base models by 1.62% to 6.14% [68]. Pretrained transformers models such as BERT for EHRs [55] and medical BERT [68] demonstrate impressive performance; however, both require a huge amount of pretraining patient data—approximately 1.6 million and 28 million samples, respectively.

Encoding diagnosis codes is a challenging task. Word2Vec is the most common method used for code-level embedding. Originally, Word2Vec was designed to capture the semantic relationships between words from large text corpora through unsupervised learning. Similarly, Word2Vec for diagnosis codes enables the encoding of meaningful relationships between medical conditions. Some models use separate training for the input data, with methods such as Word2Vec, GNNs, or transformers, while others that skip this step still require some form of embedding for codes during the training process. Consequently, it is challenging to make direct comparisons between DL models with embeddings and those without. Some studies show that learning hierarchical information through code-level embeddings can enhance the power of model prediction; for example, code-level embedding using Word2Vec can improve model performance [24]. GNNs can be a powerful tool for a hierarchical encoder with the potential to capture relationships between diagnosis codes. Many studies have demonstrated that using GNNs to embed diagnosis codes can provide effective predictions, including models such as Graph-Based Attention Model, knowledge-based attention model, Co-Attention Memory networks for diagnosis Prediction, multirelational EHR representation learning method, self-supervised graph learning framework with hyperbolic embeddings for temporal health event prediction, Sequential Diagnosis Prediction with Transformer and Ontological Representation, hypergraph-based disease prediction model using EHRs, Sequential visits and Medical Ontology, and integrated deep learning model combining long short term memory and graph neural networks. Interestingly, using multilevel representations, combining visit level and variable level, for a patient is better than single-level representation based on visit or variable alone [89].

Large language models (LLMs) have revolutionized NLP. LLMs are trained on huge amounts of text data from the internet, books, and other sources; and they can perform a wide range of language-related tasks. Recently, researchers have explored the ability of LLMs to understand medical codes; for example, a study evaluated several LLMs, including ChatGPT with GPT-3.5, ChatGPT with GPT-4, Gemini Pro, and Llama2-70b Chat, for their ability to generate correct medical codes based on code descriptions [109]. However, the study found that LLMs frequently generated incorrect codes. The findings suggest that LLMs lack an understanding of the meaning of medical codes. Therefore, it is still a challenge to use LLMs for clinical codes.

### Characteristics and Features

Generally, the performance of DL models depends on the setting of the training dataset, such as inpatient or outpatient populations. Most of the included studies (31/125, 24.8%) used models trained on the MIMIC-III dataset, which focuses on ICU admissions in the United States, with a short interval between diagnosis codes and patients considered high risk [110]. Patients in the MIMIC dataset will have more severe illnesses than a general hospital population, which would be a mix of inpatients and outpatients. The type of clinical data in the training dataset is very important for robust disease prediction. The dataset should not be focused only on specific clinical settings, such as hospital admissions or ICUs, due to data availability. However, more than half of the included studies (52/92, 57%) did not clearly report the setting of their training dataset. Moreover, a study showed that patient vital signs had a greater influence on mortality prediction in ICU settings than diagnosis codes [93]; yet, vital signs are not available in every dataset. Therefore, we suggest that it is important to provide the details of the clinical setting of the training dataset.

There may be inherent biases in how diagnosis codes are recorded in EHR data, influenced by their primary billing sources. In addition, for a single appointment, acute conditions are more likely to be recorded than chronic diseases. Any biases in the recording of diagnosis codes may transfer to the learning process of a DL algorithm. This potential transfer of bias should be carefully considered when interpreting the results. Most of the included studies (42/125, 33.6%) used datasets, such as MIMIC-III and Clinical Practice Research Datalink, that provide long-term follow-up data for large patient populations. These datasets typically include diagnosis codes for a wide range of acute and chronic medical conditions. DL studies can benefit from the availability of longer sequences of these codes, reflecting longer periods of the patient’s medical history and therefore more likely to capture both conditions.

The performance of the DL model in predicting patient outcomes depends not only on diagnosis codes but also on other features, such as demographic data [43], treatments [40,56,71], the number of visits [56], and the interval between visits [64,88]. Several studies have shown that diagnosis codes alone cannot provide the best predictive performance compared to models incorporating multiple features, such as medications, procedures, laboratory test results, demographic data, and so on [82,88,101,108]. Moreover, a study that applied BERT for EHRs has highlighted that combining diagnosis codes with factors such as age, segment, and position lead to improved precision scores compared to relying solely on diagnosis codes [55]. We propose that integrating a wide array of supplementary features, including demographic data, medication records, medical procedures, and laboratory test results, can potentially improve the performance of DL models when analyzing sequential diagnosis codes.

Many of the studies (33/84, 39%) included basic demographic data, such as gender and age, as model features. Ethnicity is another important demographic factor because it is a significant risk factor for many diseases, such as heart disease [111]. However, only a few studies (4/84, 5%) specifically described ethnicity as an input feature of their models. Most of the datasets reviewed (63/84, 75%) originated from the United States and European countries. In addition, research has shown that race and ethnicity data in EHRs are often incomplete and inaccurate, especially for minority populations [112]. These limitations can make it challenging to generalize the developed models to external settings with different patient populations and health care systems.

As diagnosis codes occur at irregular time intervals, adding time intervals as a feature can help a model to understand disease progression. Hypothetically, patients with shorter follow-up times are more likely to have severe conditions. Ablation studies, which assess model performance by removing some features, have demonstrated that integrating time intervals between patient visits can enhance predictive power. This improvement has been demonstrated across multiple models, including BiteNet [56], CATNet [88], CEHR-BERT [84], Deepr [23], DL-BERT [90], EHAN [34], and SETOR [81]. Moreover, research applying HiTANet has shown that integrating time intervals between the last visit and current visit can improve model performance [58]. A study that examined the impact of integrating time intervals as days, weeks, and months into model predictions found that using weeks as the unit yielded the best prediction performance, corresponding to the weekly follow-up pattern in real clinical practice [78]. We believe that the time interval between visits can serve as an indicator of disease progression.

Next-visit prediction is a widely applied task for evaluating DL performance in sequential diagnosis codes. However, this task carries an ROB; for example, while DL-BERT performs well in predicting the next disease based on a patient’s existing history, its precision drops—by approximately 50%—when predicting a new-onset disease that has not previously occurred in the patient’s history. This indicates that the model relies heavily on prior diagnoses rather than capturing underlying disease progression [90]. Another challenge for next-visit prediction is the issue of missing data. Sometimes, patients seek care at a different location or with a different provider, and this information may not be captured in the available data. This is a major limitation for most studies, which can be mitigated through better data linkage and information sharing across health providers.

### Evaluation, Generalizability, and Explainability

Model evaluation in health care is often complicated by class imbalance due to the nature of the medical domain, where the number of individuals with a disease is usually lower than the number of individuals without it. AUROC and *F*_1_-score are commonly used evaluation metrics in publications for health care research area and were reported by 82% (69/84) of the studies included in this review. However, *F*_1_-score focuses on positive prediction and avoid the value of true negatives. Alternatively, a study suggested using the Matthews correlation coefficient for model evaluation because it provides a more balanced assessment of positive and negative predictions [113].

Generalizability is an important issue for outcome prediction in health care. When a model is trained on a specific population, it will only perform well on patients with similar characteristics. The evaluation should include both internal and external validation. Assessing performance on different data distributions is crucial for outcome prediction [19,114]. Predictive performance degrades when models are tested on out-of-distribution years [103]; however, only a small proportion of the included studies (7/84, 8%) validated their models using data outside of their training distribution. Validating models across different settings—demographic, temporal, and geographic—is very important for real-world applications.

While DL models can yield a good performance in outcome prediction, they are frequently regarded as black box models, lacking the explainability needed to understand their internal processes and the main contributing factor for a given prediction. This lack of explainability—when the underlying reasoning is unclear—is a significant concern because it may reduce trust in predictions among health care workers and complicate the explanation of clinical decisions to patients. In recent years, researchers have focused on explainability. Shapley additive explanations (SHAP) was introduced to understand model predictions [115]. SHAP can be used to explain the decisions of LSTM models and transformers; for instance, SHAP values can explain meaningful medical codes to predict the risk of opioid overdose with 11 codes related to medications and 2 codes related to mental disorders [102]. A study introduced a tool to visualize multihead self-attention in transformer models, which can show the relationship between 2 sentences [116]. Some studies used this tool to show disease relationship between 2 sequences of diagnosis codes [55,67]. We propose that explainable artificial intelligence should be included in research studies in this area.

### Limitations

This review has several limitations. First, while our search was effective in capturing a significant proportion of the papers in this research area, it is important to note that variations in search terms may have led to the unintentional exclusion of other relevant studies, which is why we conducted a manual search to identify additional relevant articles. Second, the included studies exhibited heterogeneity, which made it difficult to compare the studies and conduct a meta-analysis. There was variability in input features and prediction tasks. Moreover, model evaluation metrics were reported differently. Although many of the included studies (57/84, 68%) introduced single, novel DL-based models, some studies (27/84, 32%) applied multiple standard models for outcome prediction. In such cases, we considered only the best-performing DL model in each study. Finally, it is important to note that PROBAST was not originally designed for evaluating DL models, and certain questions in the analysis domain are not suitable for these models.

### Recommendations and Future Work

On the basis of the findings of this review, our main recommendation for future studies using DL alongside sequential diagnostic patient information is to ensure that the generalizability of the developed models is tested on independent datasets. This will significantly reduce the ROB in key findings. Given the aforementioned limitations, we also think that studies should apply a more specific ROB assessment tool to DL models because not all questions in the current PROBAST assessment framework are suitable.

### Conclusions

The application of DL for advanced modeling of sequential medical codes has demonstrated remarkable promise in predicting patient outcomes. The main limitation of this study was the heterogeneity of methods and outcomes. However, our analysis found that using multiple types of features, integrating time intervals, and including larger sample sizes were generally related to an improved predictive performance in the included studies. This review also highlights that very few studies (7/84, 8%) reported on the challenges related to generalizability and almost half (38/84, 45%) of the studies reported challenges related to explainability of DL models. Addressing these shortcomings will be instrumental in unlocking the full potential of DL for enhancing health care outcomes and patient care.

## Appendix

Multimedia Appendix 1 The PRISMA 2020 checklist.

Multimedia Appendix 2 Additional details of included studies.

Multimedia Appendix 3 Additional figures.

## Abbreviations

AUROC: area under the receiver operating characteristic curve
BERT: bidirectional encoder representations from transformers
CNN: convolutional neural network
DL: deep learning
EHR: electronic health record
ICD-10: International Classification of Diseases, Tenth Revision
ICU: intensive care unit
LLM: large language model
LSTM: long short-term memory
MIMIC: Medical Information Mart for Intensive Care
ML: machine learning
NLP: natural language processing
PRISMA: Preferred Reporting Items for Systematic Reviews and Meta-Analyses
PROBAST: Prediction Model Study Risk of Bias Assessment Tool
RNN: recurrent neural network
ROB: risk of bias
SHAP: Shapley additive explanations

## References

[1] Huda A, Castaño A, Niyogi A, Schumacher J, Stewart M, Bruno M, Hu M, Ahmad FS, Deo RC, Shah SJ (2021). A machine learning model for identifying patients at risk for wild-type transthyretin amyloid cardiomyopathy. Nat Commun.

[2] Johnson AE, Bulgarelli L, Shen L, Gayles A, Shammout A, Horng S, Pollard TJ, Hao S, Moody B, Gow B, Lehman LW, Celi LA, Mark RG (2023). MIMIC-IV, a freely accessible electronic health record dataset. Sci Data.

[3] Pivovarov R, Albers DJ, Sepulveda JL, Elhadad N (2014). Identifying and mitigating biases in EHR laboratory tests. J Biomed Inform.

[4] Hripcsak G, Albers DJ, Perotte A (2015). Parameterizing time in electronic health record studies. J Am Med Inform Assoc.

[5] Agniel D, Kohane IS, Weber GM (2018). Biases in electronic health record data due to processes within the healthcare system: retrospective observational study. BMJ.

[6] Wu S, Roberts K, Datta S, Du J, Ji Z, Si Y, Soni S, Wang Q, Wei Q, Xiang Y, Zhao B, Xu H (2020). Deep learning in clinical natural language processing: a methodical review. J Am Med Inform Assoc.

[7] Zhou SK, Greenspan H, Davatzikos C, Duncan JS, van Ginneken B, Madabhushi A, Prince JL, Rueckert D, Summers RM (2021). A review of deep learning in medical imaging: imaging traits, technology trends, case studies with progress highlights, and future promises. Proc IEEE Inst Electr Electron Eng.

[8] Rim B, Sung NJ, Min S, Hong M (2020). Deep learning in physiological signal data: a survey. Sensors (Basel).

[9] Xiao Y, Wu J, Lin Z, Zhao X (2018). A semi-supervised deep learning method based on stacked sparse auto-encoder for cancer prediction using RNA-seq data. Comput Methods Programs Biomed.

[10] Zhou J, Cui G, Hu S, Zhang Z, Yang C, Liu Z, Wang L, Li C, Sun M (2020). Graph neural networks: a review of methods and applications. AI Open.

[11] Thorsen-Meyer HC, Nielsen AB, Nielsen AP, Kaas-Hansen BS, Toft P, Schierbeck J, Strøm T, Chmura PJ, Heimann M, Dybdahl L, Spangsege L, Hulsen P, Belling K, Brunak S, Perner A (2020). Dynamic and explainable machine learning prediction of mortality in patients in the intensive care unit: a retrospective study of high-frequency data in electronic patient records. Lancet Digit Health.

[12] Sarker IH (2021). Deep learning: a comprehensive overview on techniques, taxonomy, applications and research directions. SN Comput Sci.

[13] Xiao C, Choi E, Sun J (2018). Opportunities and challenges in developing deep learning models using electronic health records data: a systematic review. J Am Med Inform Assoc.

[14] Si Y, Du J, Li Z, Jiang X, Miller T, Wang F, Jim Zheng W, Roberts K (2021). Deep representation learning of patient data from Electronic Health Records (EHR): a systematic review. J Biomed Inform.

[15] Xie F, Yuan H, Ning Y, Ong ME, Feng M, Hsu W, Chakraborty B, Liu N (2022). Deep learning for temporal data representation in electronic health records: a systematic review of challenges and methodologies. J Biomed Inform.

[16] Carrasco-Ribelles LA, Llanes-Jurado J, Gallego-Moll C, Cabrera-Bean M, Monteagudo-Zaragoza M, Violán C, Zabaleta-Del-Olmo E (2023). Prediction models using artificial intelligence and longitudinal data from electronic health records: a systematic methodological review. J Am Med Inform Assoc.

[17] Miller SA, Forrest JL (2001). Enhancing your practice through evidence-based decision making: PICO, learning how to ask good questions. J Evid Based Dent Pract.

[18] Page MJ, McKenzie JE, Bossuyt PM, Boutron I, Hoffmann TC, Mulrow CD, Shamseer L, Tetzlaff JM, Akl EA, Brennan SE, Chou R, Glanville J, Grimshaw JM, Hróbjartsson A, Lalu MM, Li T, Loder EW, Mayo-Wilson E, McDonald S, McGuinness LA, Stewart LA, Thomas J, Tricco AC, Welch VA, Whiting P, Moher D (2021). The PRISMA 2020 statement: an updated guideline for reporting systematic reviews. BMJ.

[19] de Hond AA, Shah VB, Kant IM, Van Calster B, Steyerberg EW, Hernandez-Boussard T (2023). Perspectives on validation of clinical predictive algorithms. NPJ Digit Med.

[20] Wolff RF, Moons KG, Riley RD, Whiting PF, Westwood M, Collins GS, Reitsma JB, Kleijnen J, Mallett S (2019). PROBAST: a tool to assess the risk of bias and applicability of prediction model studies. Ann Intern Med.

[21] Landis JR, Koch GG (1977). The measurement of observer agreement for categorical data. Biometrics.

[22] Miotto R, Li L, Kidd BA, Dudley JT (2016). Deep patient: an unsupervised representation to predict the future of patients from the electronic health records. Sci Rep.

[23] Nguyen P, Tran T, Wickramasinghe N, Venkatesh S Deepr: a convolutional net for medical records. arXiv.

[24] Choi E, Bahadori MT, Schuetz A, Stewart WF, Sun J (2016). Doctor AI: predicting clinical events via recurrent neural networks. JMLR Workshop Conf Proc.

[25] Pham T, Tran T, Phung D, Venkatesh S DeepCare: a deep dynamic memory model for predictive medicine. arXiv.

[26] Choi E, Bahadori MT, Kulas JA, Schuetz A, Stewart WF, Sun J RETAIN: an interpretable predictive model for healthcare using reverse time attention mechanism. arXiv.

[27] Ma F, Chitta R, Zhou J, You Q, Sun T, Gao J Dipole: diagnosis prediction in healthcare via attention-based bidirectional recurrent neural networks. arXiv.

[28] Choi E, Bahadori MT, Song L, Stewart WF, Sun J (2017). GRAM: graph-based attention model for healthcare representation learning. KDD.

[29] Sha Y, Wang MD (2017). Interpretable predictions of clinical outcomes with an attention-based recurrent neural network. ACM BCB.

[30] Choi E, Schuetz A, Stewart WF, Sun J (2017). Using recurrent neural network models for early detection of heart failure onset. J Am Med Inform Assoc.

[31] Suo Q, Ma F, Yuan Y, Huai M, Zhong W, Zhang A, Gao J (2017). Personalized disease prediction using a CNN-based similarity learning method. Proceedings of the IEEE International Conference on Bioinformatics and Biomedicine.

[32] Amirkhan R, Hoogendoorn M, Numans ME, Moons L (2017). Using recurrent neural networks to predict colorectal cancer among patients. Proceedings of the IEEE Symposium Series on Computational Intelligence.

[33] Lei L, Zhou Y, Zhai J, Zhang L, Fang Z, He P, Gao J (2018). An effective patient representation learning for time-series prediction tasks based on EHRs. Proceedings of the IEEE International Conference on Bioinformatics and Biomedicine.

[34] Park S, Kim YJ, Kim JW, Park JJ, Ryu B, Ha JW (2018). Interpretable prediction of vascular diseases from electronic health records via deep attention networks. Proceedings of the IEEE 18th International Conference on Bioinformatics and Bioengineering.

[35] Bai T, Egleston BL, Zhang S, Vucetic S (2018). Interpretable representation learning for healthcare via capturing disease progression through time. KDD.

[36] Choi E, Xiao C, Stewart WF, Sun J (2018). MiME: multilevel medical embedding of electronic health records for predictive healthcare. Proceedings of the 32nd International Conference on Neural Information Processing Systems.

[37] Qiao Z, Zhao S, Xiao C, Li X, Qin Y, Wang F (2018). Pairwise-ranking based collaborative recurrent neural networks for clinical event prediction. Proceedings of the Twenty-Seventh International Joint Conference on Artificial Intelligence.

[38] Wang WW, Li H, Cui L, Hong X, Yan Z (2018). Predicting clinical visits using recurrent neural networks and demographic information. Proceedings of the IEEE 22nd International Conference on Computer Supported Cooperative Work in Design.

[39] Ma F, You Q, Xiao H, Chitta R, Zhou J, Gao J (2018). KAME: knowledge-based attention model for diagnosis prediction in healthcare. Proceedings of the 27th ACM International Conference on Information and Knowledge Management.

[40] Zhang J, Kowsari K, Harrison JH, Lobo JM, Barnes LE (2018). Patient2Vec: a personalized interpretable deep representation of the longitudinal electronic health record. IEEE Access.

[41] Jin B, Che C, Liu Z, Zhang S, Yin X, Wei X (2018). Predicting the risk of heart failure with EHR sequential data modeling. IEEE Access.

[42] Guo W, Ge W, Cui L, Li H, Kong L (2019). An interpretable disease onset predictive model using crossover attention mechanism from electronic health records. IEEE Access.

[43] Lin YW, Zhou Y, Faghri F, Shaw MJ, Campbell RH (2019). Analysis and prediction of unplanned intensive care unit readmission using recurrent neural networks with long short-term memory. PLoS One.

[44] Wang W, Guo C, Xu J, Liu A (2019). Bi-dimensional representation of patients for diagnosis prediction. Proceedings of the IEEE 43rd Annual Computer Software and Applications Conference.

[45] Gao J, Wang X, Wang Y, Yang Z, Gao J, Wang J, Tang W, Xie X (2019). CAMP: co-attention memory networks for diagnosis prediction in healthcare. Proceedings of the 2019 IEEE International Conference on Data Mining.

[46] Ma F, Wang Y, Xiao H, Yuan Y, Chitta R, Zhou J, Gao J (2019). Incorporating medical code descriptions for diagnosis prediction in healthcare. BMC Med Inform Decis Mak.

[47] AlSaad R, Malluhi Q, Janahi I, Boughorbel S (2019). Interpreting patient-specific risk prediction using contextual decomposition of BiLSTMs: application to children with asthma. BMC Med Inform Decis Mak.

[48] Zhang XS, Tang F, Dodge HH, Zhou J, Wang F (2019). MetaPred: meta-learning for clinical risk prediction with limited patient electronic health records. KDD.

[49] Ashfaq A, Sant'Anna A, Lingman M, Nowaczyk S (2019). Readmission prediction using deep learning on electronic health records. J Biomed Inform.

[50] Ruan T, Lei L, Zhou Y, Zhai J, Zhang L, He P, Gao J (2019). Representation learning for clinical time series prediction tasks in electronic health records. BMC Med Inform Decis Mak.

[51] Huang Y, Yang X, Xu C Time-guided high-order attention model of longitudinal heterogeneous healthcare data. arXiv.

[52] Xiang Y, Xu J, Si Y, Li Z, Rasmy L, Zhou Y, Tiryaki F, Li F, Zhang Y, Wu Y, Jiang X, Zheng WJ, Zhi D, Tao C, Xu H (2019). Time-sensitive clinical concept embeddings learned from large electronic health records. BMC Med Inform Decis Mak.

[53] Gupta M, Phan TL, Bunnell T, Beheshti R Obesity prediction with EHR data: a deep learning approach with interpretable elements. arXiv.

[54] Shi P, Hou F, Zheng X, Yuan F (2020). Analysis of electronic health records based on long short‐term memory. Concurr Comput.

[55] Li Y, Rao S, Solares JR, Hassaine A, Ramakrishnan R, Canoy D, Zhu Y, Rahimi K, Salimi-Khorshidi G (2020). BEHRT: transformer for electronic health records. Sci Rep.

[56] Peng X, Long G, Shen T, Wang S, Jiang J, Zhang C (2020). BiteNet: bidirectional temporal encoder network to predict medical outcomes. Proceedings of the 2020 IEEE International Conference on Data Mining.

[57] Almog YA, Rai A, Zhang P, Moulaison A, Powell R, Mishra A, Weinberg K, Hamilton C, Oates M, McCloskey E, Cummings SR (2020). Deep learning with electronic health records for short-term fracture risk identification: crystal bone algorithm development and validation. J Med Internet Res.

[58] Luo J, Ye M, Xiao C, Ma F (2020). HiTANet: hierarchical time-aware attention networks for risk prediction on electronic health records. Proceedings of the 26th ACM SIGKDD International Conference on Knowledge Discovery & Data Mining.

[59] Zhang X, Qian B, Cao S, Li Y, Chen H, Zheng Y, Davidson I (2020). INPREM: an interpretable and trustworthy predictive model for healthcare. Proceedings of the 26th ACM SIGKDD International Conference on Knowledge Discovery & Data Mining.

[60] Rongali S, Rose AJ, McManus DD, Bajracharya AS, Kapoor A, Granillo E, Yu H (2020). Learning latent space representations to predict patient outcomes: model development and validation. J Med Internet Res.

[61] Ye M, Luo J, Xiao C, Ma F (2020). LSAN: modeling long-term dependencies and short-term correlations with hierarchical attention for risk prediction. Proceedings of the 29th ACM International Conference on Information & Knowledge Management.

[62] Zeng X, Feng Y, Moosavinasab S, Lin D, Lin S, Liu C (2020). Multilevel self-attention model and its use on medical risk prediction. Pac Symp Biocomput.

[63] An Y, Mao Y, Zhang L, Jin B, Xiao K, Wei X, Yan J (2020). RAHM: relation augmented hierarchical multi-task learning framework for reasonable medication stocking. J Biomed Inform.

[64] Kabeshova A, Yu Y, Lukacs B, Bacry E, Gaïffas S (2020). ZiMM: a deep learning model for long term and blurry relapses with non-clinical claims data. J Biomed Inform.

[65] Darabi S, Kachuee M, Fazeli S, Sarrafzadeh M (2020). TAPER: Time-Aware Patient EHR Representation. IEEE J Biomed Health Inform.

[66] An Y, Huang N, Chen X, Wu F, Wang J (2021). High-risk prediction of cardiovascular diseases via attention-based deep neural networks. IEEE/ACM Trans Comput Biol Bioinform.

[67] Meng Y, Speier W, Ong MK, Arnold CW (2021). Bidirectional representation learning from transformers using multimodal electronic health record data to predict depression. IEEE J Biomed Health Inform.

[68] Rasmy L, Xiang Y, Xie Z, Tao C, Zhi D (2021). Med-BERT: pretrained contextualized embeddings on large-scale structured electronic health records for disease prediction. NPJ Digit Med.

[69] Ju R, Zhou P, Wen S, Wei W, Xue Y, Huang X, Yang X (2021). 3D-CNN-SPP: a patient risk prediction system from electronic health records via 3D CNN and spatial pyramid pooling. IEEE Trans Emerg Top Comput Intell.

[70] Harerimana G, Kim JW, Jang B (2021). A deep attention model to forecast the Length Of Stay and the in-hospital mortality right on admission from ICD codes and demographic data. J Biomed Inform.

[71] Ningrum DN, Kung WM, Tzeng IS, Yuan SP, Wu CC, Huang CY, Muhtar MS, Nguyen PA, Li JY, Wang YC (2021). A deep learning model to predict knee osteoarthritis based on nonimage longitudinal medical record. J Multidiscip Healthc.

[72] Florez AY, Scabora L, Eler DM, Rodrigues JF (2021). APEHR: automated Prognosis in electronic health records using multi-head self-attention. Proceedings of the IEEE 34th International Symposium on Computer-Based Medical Systems.

[73] Pham TH, Yin C, Mehta L, Zhang X, Zhang P (2021). Cardiac complication risk profiling for cancer survivors via multi-view multi-task learning. Proceedings of the IEEE International Conference on Data Mining.

[74] Boursalie O, Samavi R, Doyle TE (2021). Decoder transformer for temporally-embedded health outcome predictions. Proceedings of the 20th IEEE International Conference on Machine Learning and Applications.

[75] Dong X, Deng J, Rashidian S, Abell-Hart K, Hou W, Rosenthal RN, Saltz M, Saltz JH, Wang F (2021). Identifying risk of opioid use disorder for patients taking opioid medications with deep learning. J Am Med Inform Assoc.

[76] Kwak H, Chang J, Choe B, Park S, Jung K (2021). Interpretable disease prediction using heterogeneous patient records with self-attentive fusion encoder. J Am Med Inform Assoc.

[77] Sun C, Dui H, Li H (2021). Interpretable time-aware and co-occurrence-aware network for medical prediction. BMC Med Inform Decis Mak.

[78] Men L, Ilk N, Tang X, Liu Y (2021). Multi-disease prediction using LSTM recurrent neural networks. Expert Syst Appl.

[79] Shi Y, Guo Y, Wu H, Li J, Li X (2021). Multi-relational EHR representation learning with infusing information of diagnosis and medication. Proceedings of the IEEE 45th Annual Computers, Software, and Applications Conference.

[80] Lu C, Reddy CK, Ning Y Self-supervised graph learning with hyperbolic embedding for temporal health event prediction. arXiv.

[81] Peng X, Long G, Shen T, Wang S, Jiang J (2021). Sequential diagnosis prediction with transformer and ontological representation. Proceedings of the IEEE International Conference on Data Mining.

[82] An Y, Tang K, Wang J (2021). Time-aware multi-type data fusion representation learning framework for risk prediction of cardiovascular diseases. IEEE/ACM Trans Comput Biol Bioinform.

[83] Poulain R, Gupta M, Foraker R, Beheshti R (2021). Transformer-based multi-target regression on electronic health records for primordial prevention of cardiovascular disease. Proceedings (IEEE Int Conf Bioinformatics Biomed).

[84] Pang C, Jiang X, Kalluri KS, Spotnitz M, Chen R, Perotte A, Natarajan K CEHR-BERT: incorporating temporal information from structured EHR data to improve prediction tasks. arXiv.

[85] Rao S, Li Y, Ramakrishnan R, Hassaine A, Canoy D, Cleland J, Lukasiewicz T, Salimi-Khorshidi G, Rahimi K (2022). An explainable transformer-based deep learning model for the prediction of incident heart failure. IEEE J Biomed Health Inform.

[86] Du J, Zeng D, Li Z, Liu J, Lv M, Chen L, Zhang D, Ji S (2021). An interpretable outcome prediction model based on electronic health records and hierarchical attention. Int J Intell Syst.

[87] De Barros PH, Rodrigues JF (2022). AttentionHCare: advances on computer-aided medical prognosis using attention-based neural networks. Proceedings of the International Joint Conference on Neural Networks.

[88] Liu S, Wang X, Xiang Y, Xu H, Wang H, Tang B (2022). CATNet: cross-event attention-based time-aware network for medical event prediction. Artif Intell Med.

[89] Yang F, Zhang J, Chen W, Lai Y, Wang Y, Zou Q (2022). DeepMPM: a mortality risk prediction model using longitudinal EHR data. BMC Bioinformatics.

[90] Chen X, Lin J, An Y (2022). DL-BERT: a time-aware double-level BERT-style model with pre-training for disease prediction. Proceedings of the IEEE International Conference on Big Data.

[91] Sun Z, Yang X, Feng Z, Xu T, Fan X, Tian J (2022). EHR2HG: modeling of EHRs data based on hypergraphs for disease prediction. Proceedings of the IEEE International Conference on Bioinformatics and Biomedicine.

[92] Yu F, Cui L, Cao Y, Zhu F, Xu Y, Liu N (2022). Feature-guided logical perception network for health risk prediction. Proceedings of the IEEE International Conference on Bioinformatics and Biomedicine.

[93] Niu K, Lu Y, Peng X, Zeng J (2022). Fusion of sequential visits and medical ontology for mortality prediction. J Biomed Inform.

[94] AlSaad R, Malluhi Q, Janahi I, Boughorbel S (2022). Predicting emergency department utilization among children with asthma using deep learning models. Healthc Anal.

[95] Gerrard L, Peng X, Clarke A, Schlegel C, Jiang J (2021). Predicting outcomes for cancer patients with transformer-based multi-task learning. Proceedings of the Advances in Artificial Intelligence.

[96] AlSaad R, Malluhi Q, Boughorbel S (2022). PredictPTB: an interpretable preterm birth prediction model using attention-based recurrent neural networks. BioData Min.

[97] Ramchand S, Tsang G, Cole D, Xie X (2022). RetainEXT: enhancing rare event detection and improving interpretability of health records using temporal neural networks. Proceedings of the IEEE-EMBS International Conference on Biomedical and Health Informatics.

[98] Andjelkovic J, Ljubic B, Hai AA, Stanojevic M, Pavlovski M, Diaz W, Obradovic Z (2022). Sequential machine learning in prediction of common cancers. Informatics Med Unlocked.

[99] Yu F, Cui L, Cao Y, Liu N, Huang W, Xu Y (2022). Similarity-aware collaborative learning for patient outcome prediction. Proceedings of the Database Systems for Advanced Applications.

[100] Li Y, Salimi-Khorshidi G, Rao S, Canoy D, Hassaine A, Lukasiewicz T, Rahimi K, Mamouei M (2022). Validation of risk prediction models applied to longitudinal electronic health record data for the prediction of major cardiovascular events in the presence of data shifts. Eur Heart J Digit Health.

[101] Li Y, Mamouei M, Salimi-Khorshidi G, Rao S, Hassaine A, Canoy D, Lukasiewicz T, Rahimi K (2023). Hi-BEHRT: hierarchical transformer-based model for accurate prediction of clinical events using multimodal longitudinal electronic health records. IEEE J Biomed Health Inform.

[102] Dong X, Wong R, Lyu W, Abell-Hart K, Deng J, Liu Y, Hajagos JG, Rosenthal RN, Chen C, Wang F (2023). An integrated LSTM-HeteroRGNN model for interpretable opioid overdose risk prediction. Artif Intell Med.

[103] Guo LL, Steinberg E, Fleming SL, Posada J, Lemmon J, Pfohl SR, Shah N, Fries J, Sung L (2023). EHR foundation models improve robustness in the presence of temporal distribution shift. Sci Rep.

[104] Liang Y, Guo C (2023). Heart failure disease prediction and stratification with temporal electronic health records data using patient representation. Biocybern Biomed Eng.

[105] Lee LT, Yang HC, Nguyen PA, Muhtar MS, Li YC (2023). Machine learning approaches for predicting psoriatic arthritis risk using electronic medical records: population-based study. J Med Internet Res.

[106] Devlin J, Chang MW, Lee K, Toutanova K BERT: pre-training of deep bidirectional transformers for language understanding. arXiv.

[107] ChatGPT homepage. ChatGPT.

[108] Zhang Y, Wang H, Zhang D, Wang D (2019). DeepRisk: a deep transfer learning approach to migratable traffic risk estimation in intelligent transportation using social sensing. Proceedings of the 2019 15th International Conference on Distributed Computing in Sensor Systems.

[109] Soroush A, Glicksberg BS, Zimlichman E, Barash Y, Freeman R, Charney AW, Nadkarni GN, Klang E (2024). Large language models are poor medical coders — benchmarking of medical code querying. NEJM AI.

[110] Johnson AE, Pollard TJ, Shen LW, Lehman LH, Feng M, Ghassemi M, Moody B, Szolovits P, Celi LA, Mark RG (2016). MIMIC-III, a freely accessible critical care database. Sci Data.

[111] Post WS, Watson KE, Hansen S, Folsom AR, Szklo M, Shea S, Barr RG, Burke G, Bertoni AG, Allen N, Pankow JS, Lima JA, Rotter JI, Kaufman JD, Johnson WC, Kronmal RA, Diez-Roux AV, McClelland RL (2022). Racial and ethnic differences in all-cause and cardiovascular disease mortality: the MESA study. Circulation.

[112] Yemane L, Mateo CM, Desai AN (2024). Race and ethnicity data in electronic health records-striving for clarity. JAMA Netw Open.

[113] Chicco D, Jurman G (2020). The advantages of the Matthews correlation coefficient (MCC) over F1 score and accuracy in binary classification evaluation. BMC Genomics.

[114] Guo LL, Pfohl SR, Fries J, Johnson AE, Posada J, Aftandilian C, Shah N, Sung L (2022). Evaluation of domain generalization and adaptation on improving model robustness to temporal dataset shift in clinical medicine. Sci Rep.

[115] Lundberg S, Lee SI A unified approach to interpreting model predictions. arXiv.

[116] Vig J Visualizing attention in transformer-based language representation models. arXiv.

