# Supplementary material for: Enhancing Patient Outcome Prediction Through Deep Learning With Sequential Diagnosis Codes From Structured Electronic Health Record Data: Systematic Review
Source: J Med Internet Res. 2025 Mar 18;27:e57358. doi: 10.2196/57358 (PMC11962322; doi:10.2196/57358)
Supplement: Multimedia Appendix 3 [file jmir_v27i1e57358_app3.docx]

**Supplement for:** **Enhancing Patient Outcome Prediction through Deep Learning with Sequential Diagnosis Codes from Structured EHR: A systematic review**

**Supplementary Figures**

Supplementary figure 1 - The distribution of sample size 2

Supplementary figure 2 - Data sources 3

Supplementary figure 3 - Additional features 4

Supplementary figure 4 - Model performance 5


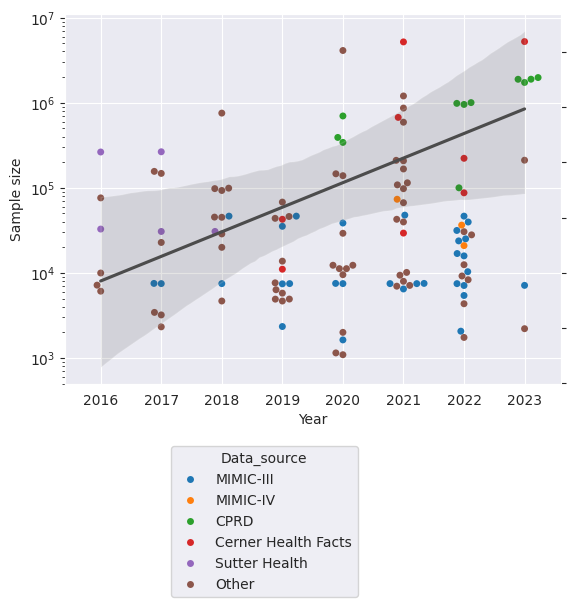


**Fig 1.** The distribution of sample size according to publication year


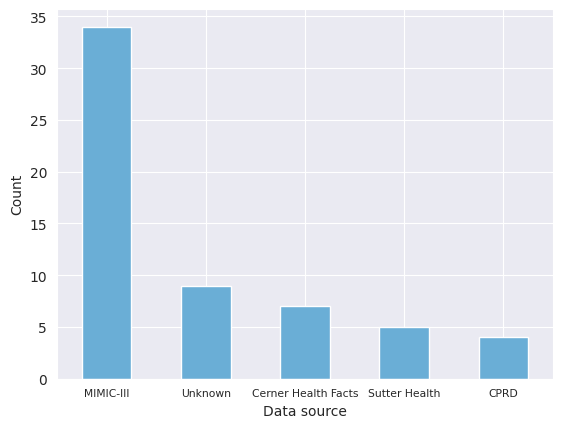


**Fig 2.** Top 5 data sources for deep learning training

**
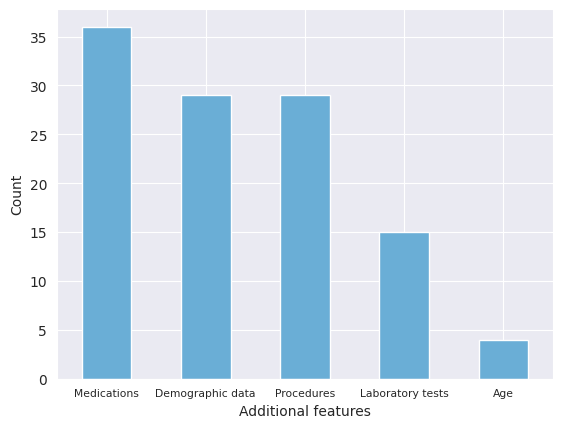
**

**Fig 3.** Top 5 additional features for deep learning training

**
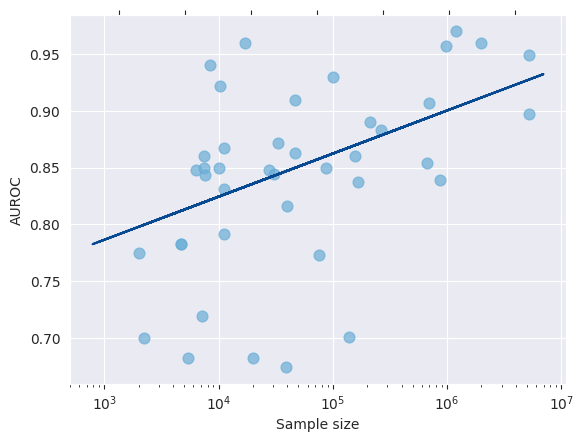
**

**
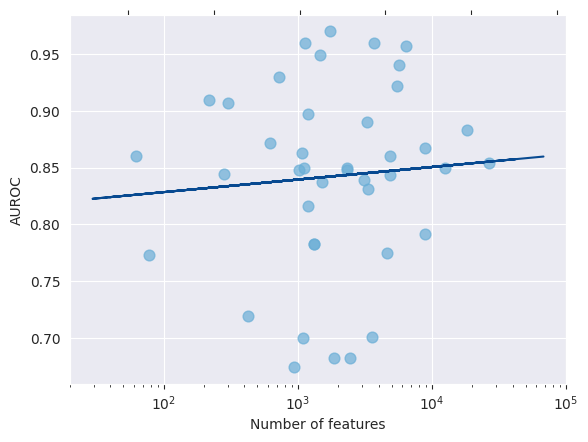
**

**
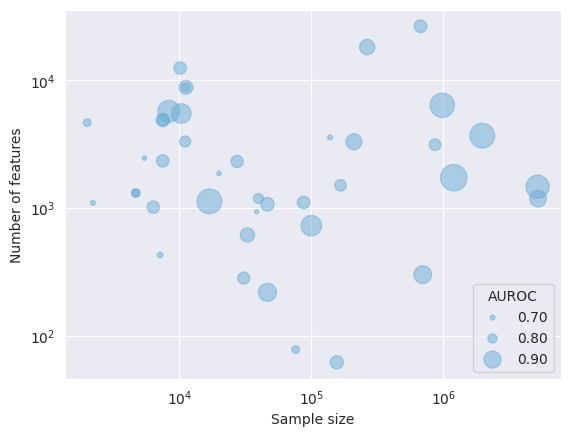
**

**Fig 4.** Forty-one studies report AUROC as their model performance. Overview of sample sizes and number of features with point size proportional to AUROC (upper). Relationship between sample size and AUROC (middle). Relationship between number of features and AUROC (lower) The number of features is the combination between the number of medical codes and additional information (demographic data, medications, procedures, etc.)
